# Propofol-Based Sedation Does Not Increase Rate of Complication during Percutaneous Endoscopic Gastrostomy Procedure

**DOI:** 10.1155/2011/134819

**Published:** 2010-08-03

**Authors:** Somchai Amornyotin, Wiyada Chalayonnavin, Siriporn Kongphlay

**Affiliations:** Department of Anesthesiology, Siriraj GI Endoscopy Center, Faculty of Medicine Siriraj Hospital, Mahidol University, Bangkok 10700, Thailand

## Abstract

*Objectives*. To evaluate and compare the complication rate of sedation with or without propofol regimen for percutaneous endoscopic gastrostomy (PEG) in a hospital in Thailand. *Subjects and Methods*. A total of 198 patients underwent PEG procedures by using intravenous sedation (IVS) from Siriraj Hospital, Thailand from August 2006 to January 2009. The primary outcome variable was the overall complication rate. The secondary outcome variables were sedation and procedure related complications, and mortality rate. *Results*. After matching ASA physical status and indications of procedure, there were 92 PEG procedures in propofol based sedation group (A) and 20 PEG procedures in non-propofol based sedation group (B). All sedation was given by residents or anesthetic nurses directly supervised by staff anesthesiologist in the endoscopy room. There were no significant differences in patients' characteristics, sedation time, indication, complications, anesthetic personnel and mortality rate between the two groups. All complications were easily treated, with no adverse sequelae. Mean dose of fentanyl and midazolam in group A was significantly lower than in group B. *Conclusion*. Propofol-based sedation does not increase rate of complication during PEG procedure. Additionally, IVS of PEG procedure is relatively safe and effective when performed by physicians in training. Serious complications are none.

## 1. Introduction

Percutaneous endoscopic gastrostomy (PEG) has become the procedure of choice for enteral feedings in patients with a functioning gastrointestinal tract who need long-term enteral feeding, when oral access is impossible [1, 2]. PEG has replaced the surgical gastrostomy procedure because of its lower cost and shorter recovery time. Many patients requiring PEG are older, frail, and malnourished and have a significant comorbidity. PEG insertion is an invasive procedure requiring both endoscopy and sedation. It usually carries a risk of high mortality rates in the early postinsertion period, with 30-day mortality rates varying between 4% and 26% [3]. Furthermore, there is a substantial risk of morbidity, especially from sedation and/or anesthesia [4]. 

 Anesthesia consultation before the procedure is needed. Fluid and electrolyte disorders should be corrected and any infection treated. Antibiotic prophylaxis is recommended due to the infection risks. Ideally, PEG should be performed in an operating room. In practice, however, most procedures are performed in the endoscopy room, with special precautions. The type of anesthesia used is decided according to the patient's medical condition and the anesthesiologist's preference. Intravenous sedation (IVS) can be used, but to assure better patient comfort during this complicated procedure, short-term deep sedation is preferred.

 We conducted a retrospective study to discover whether there is a difference in the incidence of complication rate between patients who received PEG procedure with or without propofol-based sedation and to evaluate the safety of IVS when sedated by anesthetic personnel from the World Gastroenterology Organization (WGO) Endoscopy Training Center in Thailand.

## 2. Materials and Method

### 2.1. Patients

A total of 279 consecutive patients from Siriraj Hospital, Bangkok, Thailand who underwent PEG procedures from August 2006 to January 2009 were eligible for the study. Of these, 198 patients underwent PEG procedures by using intravenous sedation (IVS). Inclusion criteria were age ≥18 and PEG procedures performed using IVS technique. Exclusion criteria were patients younger than 18 years, procedures performed in the intensive care units, procedures performed without sedation, or procedures performed under monitored anesthesia care and general anesthesia.

### 2.2. Study Design

This study is a retrospective descriptive study. All patients were classified into two groups according to the type of IVS technique. In group A, PEG was done by using propofol-based IVS technique. In group B, PEG was performed with non-propofol-based IVS technique. The primary outcome variable of the study was the overall complication rate during and immediately after procedure. The secondary outcome variables were sedation- and procedure related complications during and immediately after procedure and mortality rate.

### 2.3. Assessment of Complication Rate

After PEG procedure, all patients were observed in the recovery room at least two hours before discharged to ward. Additionally, all patients were admitted in the ward to rule out post-PEG complications at least one day after the procedure. We did not call each patient at the thirtieth day after the procedure. Overall complication rate in both groups was recorded. Additionally, sedation- and procedure related complication and mortality rate in the two groups were also assessed.

### 2.4. Sedation-Related Complications

All Sedation-related complications were recorded. Sedation-related complications were defined as follows: hypertension or hypotension (increase or decrease in blood pressure by 20% from baseline and above or below normal for age); tachycardia or bradycardia (increase or decrease in heart rate by 20% from baseline and above or below normal for age); any cardiac arrhythmias; hypoxia (oxygen desaturation, SpO_2_ < 90%); airway obstruction.

### 2.5. Procedure Related Complications

Procedure related complications during and early post procedure such as bleeding, laceration or puncture of visceral organs, and PEG-site infection were recorded. We did not assess the late complications.

### 2.6. Statistical Analysis

Results were expressed as mean ± SD or percentage (%), when appropriate. Comparisons between group A and B weredoneby using Chi-square tests (for categorical variables), Chi-square tests for trend (for ordinal variables), and two-sample independent *t*-test (for continuous variables). The statistical software package SPSS for Window Version 11 (SPSS Inc., Chicago, IL) was used to analyze the data. All statistical comparisons were made at the two-sided 5% level of significance.

## 3. Results

Two hundred and seventy-nine PEG procedures were performed during the study period. Of these, 81 patients who underwent PEG procedure by using general anesthesia and monitored anesthesia care techniques were excluded. A total of 198 PEG procedures were performed by using IVS. Of these, 178 patients were classified in propofol-based sedation group and 20 patients were in non-propofol-based sedation group. After matching ASA physical status and the indications of procedure, there were 92 PEG procedures in group A and 20 PEG procedures in group B. 

Table 1 showed the characteristics of patients, duration of sedation, and indications of procedure. There were no statistically significant differences in age, gender, weight, ASA physical status, sedation time, and indication of the procedure between the two groups. 

 Cardiovascular monitoring, including blood pressure measurements, electrocardiogram, heart rate, and oxygen saturation, was performed. No premedications were used before the procedure. All patients were oxygenated with 100% O_2_ via nasal canular and sedated by well-trained anesthetic personnel directly supervised by a staff anesthesiologist in the endoscopy room. Anesthetic personnel included residents in anesthesiology and anesthetic nurses who were well trained in the use of IVS technique and airway management. All sedated patients were sedated in either moderate (conscious) or deep sedation level, according to guideline of the American Society of Anesthesiologists [5]. Subsequently, all cases were concluded with the satisfactory completion of the procedure.

Table 2 demonstrated overall complication rate, sedation and procedure related complication, anesthetic personnel, and mortality rate. Overall, 23 patients (25.0%) in group A and 4 patients (20.0%) in group B experienced adverse events. In group A, the respiratory adverse events occurred in 5.4% of patients and comprised 21.7% of all adverse events, and all of these were under the care of an anesthesiologist. Interestingly, there were no respiratory adverse events in group B. Cardiovascular adverse events arose in 18.5% and 20.0% of patients in group A and B, respectively. They mainly consisted of hypotension (16.3% in group A and 10.0% in group B). One patient in group B developed hypertension and tachycardia but none in group A. No procedures were aborted as a result of insufficient sedation or complications of sedation. In addition, only one patient in group A developed bleeding after the procedure. In both groups, IVS was mainly employed by the residents, and mortality rate was none. However, there were no significant differences in overall complication rate, sedation and procedure related complication, anesthetic personnel, and mortality rate between the two groups. 

Table 3 showed the sedative agents used in both groups. Of these, fentanyl and midazolam were frequently used in both groups. Mean dose of fentanyl and midazolam in group A was significantly lower than in group B (*P* = .012 and <.001, resp.). However, there was not statistical difference in the mean dose of ketamine between the two groups (*P* = .333). 

 Hemodynamic parameters including systolic and diastolic blood pressure, heart rate, and oxygen saturation were demonstrated in Table 4. There were not significant differences between the groups in hemodynamic parameters at baseline, insertion of endoscope, and at 15, 25, and 30 minutes after scope insertion. However, mean systolic blood pressure at 5 and 10 minutes after scope insertion, as well as mean diastolic blood pressure at 5 and 20 minutes, after scope insertion in the propofol-based group was significantly lower than in the non-propofol-based group. In addition, mean heart rate at 20 minutes after scope insertion in the non-propofol-based group was significantly higher than in the propofol-based group. Oxygen saturation in both groups was over 99% through out the study period.

## 4. Discussion

PEG has rapidly replaced surgical gastrostomy as the procedure of choice in virtual patients requiring long-term enteral nutrition. Increasing numbers of patients are being referred for PEG placement. PEG can be inserted in the operating room, endoscopy suite, or at the bedside using IVS. The overall success rate for PEG placement is rather consistent at 95% to 98% in all studies, regardless of technique [6–8]. Procedure-related complications are infrequent (1.5% to 4.0%) [9, 10]. However, cardiovascular complications related to sedation/analgesia are the most frequent complications of diagnostic endoscopy and PEG procedure [11–13]. 

 Our study showed that the rate of complication during PEG procedure with or without propofol-based sedation was comparable to our previous reports [12, 13]. In addition, the propofol-based sedation does not increase the complication rate in comparison to the non-propofol-based sedation (*P* = .636). However, the complication rate in this present study is markedly higher than the published study [11]. One possible explanation of this finding is that the number of PEGs underwent IVS technique has remarkably increased over the last few years. The depth of sedation in our report was moderate to deep level. Additionally, this study collected only PEG procedures by using IVS technique. In that published study [11], upper and lower gastrointestinal endoscopy procedures done with conscious (moderate) sedation technique were included the sedation-related complication rate was 0.54%. However, the previous series did not mention about the frequently used propofol-based sedation technique. The result of our study also demonstrated that the complication rate would be correlated to the depth of sedation directly. Moreover, the results of other studies [14–17] also confirmed that patients could withstand PEG procedure without sedation, and the rate of complication was fairly low in this technique. 

 PEG procedure is a minimally invasive one, with low procedure-related major complications and mortality rates [10, 18–20]. It is an essential procedure among GI abnormality treatments, even in our institution, where we observe an increase in number of these procedures every year. Therefore, it is mandatory to standardize a safe, easy, well-tolerated anesthesiological procedure, which is feasible in the GI endoscopy unit. In our previous experiences, we have noted that topical anesthesia alone is not sufficient for pain-free procedures. In contrast, general anesthesia, which may be of benefit for the patient and endoscopist comforts, may be difficult to administer, especially in comorbidity patients. In addition, the lack of experience in anesthesia care among endoscopy personnel might increase the risk of complications. 

 Propofol, combined with short-acting benzodiazepine, with or without fentanyl, has already been used in several GI endoscopic procedures. The present study shows that sedation with or without propofol is safe and well tolerated by the patient. Furthermore, it is well accepted by endoscopists. No patients enrolled in the study needed to be resuscitated during PEG procedure. All patients could be discharged to the ward within 30 minutes from the end of this procedure, and this discharge time was not correlated with age, ASA physical status, and total sedative doses.

 Patients were breathing spontaneously; however, oxygen saturation was always over 99%, and age, ASA physical status, and the combination of sedative agents did not negatively influence this parameter. Sedation is performed to ensure the patient's safety, to minimize physical discomfort or pain, to provide analgesia and procedural amnesia, to control behavior during the procedure and to return the patient to pretreatment level of consciousness. The amount of sedation required depends on the patient's physical status and age. Propofol is widely employed for anesthesia outside the OR because it is easy to use, has a good safety and efficacy profile due to its quick onset of action, rapid metabolism, and significantly shorter recovery time, and has some antiemetic effects [21–23]. Low-dose of midazolam as well as ketamine, combined with low dose fentanyl and/or propofol, did not prolong recovery time. Additionally, ketamine in the company of these agents did not produce emergence reactions or hallucinations. 

 The present study used only standard monitoring, including an assessment of blood pressure, pulse rate, respiratory rate and pulse oximetry, as well as electrocardiogram. We detected a relatively high overall rate of adverse events in both groups. This rate is higher than that commonly reported, and there may be several explanations. We used these criteria in defining adverse events: hypo/hypertension and brady/tachycardia measured as the changes of blood pressure and heart rate of more than 20% of baseline values. Hypoxia was defined as oxygen saturation <90%. Hypercapnia (ETCO_2_ > 50 mmHg) could not be detected directly in this study. Moreover, if only serious adverse events are included, the adverse event rate is 2.2% in the propofol-based group and none in the non-propofol-based group. Interestingly, we found that all respiratory-related adverse events occurred in the propofol-based group. 

 In a previous study [24], 151 high risk patients underwent PEGs (126), and direct jejunostomies (PEJs, 25) were sedated by the use of anesthesiologist-administered propofol. Minor complications occurred in 25 patients (16.6%): 13 patients (8.6%) fevers, 12 patients (7.9%) systolic blood pressure drops of >25%, and 1 patient (0.6%) oxygen desaturation <90%. Major complications occurred in 4 patients (2.6%): 3 patients (2%) aspiration pneumonias and one patient death (0.6%). We believe that the appropriate selection of patients for sedation is very important for everyday practice and will most likely reduce the rate of adverse events. Finally, the use of pulse oximetry to monitor hypoxemia is important, especially in cases when supplemental oxygen is administered.

 Data from our previous study [25] showed that satisfaction of both patient and endoscopist about sedated patients was higher than in nonsedated patients. The use of sedation was the major determinant of patient satisfaction and willingness to repeat. Among all of these benefits, it is advantageous to identify the particular factors that might encourage patients to undergo PEG procedure with sedation. Moreover, the present study showed that PEG procedures can be performed safely and effectively with a lower complication rate under propofol-based sedation. Additionally, our recent report [12] also shows that the PEG procedure done with sedation by well-trained anesthetic personnel is as safe and effective as that done with general anesthesia. In our hospital, IVS technique was extensively used for PEG procedures. However, this is not widespread in the district community hospitals.

 Limitations of this study exist. First, there is the wide range in age of the patients in our study. Drug requirements and adverse events can be related to patient's age. Second, inaccurate and incomplete documentation of certain measures, as occured with many chart reviews, also occurred in this study. Third, the limitation of monitoring, such as of end-tidal carbon dioxide, could result in a lower rate of adverse events. Overall, despite these limitations, we are, however, confident that these findings are generalizable to the practice of PEG procedure using any type of sedation. Finally, because the serious complications in our series were low, further studies in larger prospective groups of patients are therefore needed.

 In conclusion, we report the performance of the clinical efficacy of sedation with or without propofol regimen utilizing anesthesiologist or anesthetic personnel with appropriate basic monitoring for PEG procedure in a unit outside OR from a tertiary-care teaching hospital in Thailand. The findings of the present study show that propofol-based sedation does not increase rate of complication during PEG procedure. IVS of PEG procedure is relatively safe and effective when performed by physicians in training. Serious complications are none. We hope that our practice will help modeling the development of IVS for PEG procedure in the community hospitals in Thailand.

## Figures and Tables

**Table 1:** Characteristics of patients, duration of sedation, and indications of procedure (mean, SD and percentage).

	Group A	Group B	*P-* value
	(*N* = 92)	(*N* = 20)	
Age (yr) (mean, SD)	70.3 (8.5)	75.2 (10.7)	.376
Gender (%): Male	43 (46.7)	9 (45.0)	.888
Female	49 (53.3)	11 (55.0)	
Weight (kg) (mean, SD)	49.6 (4.8)	48.1 (6.4)	.107
ASA physical status (%): I-II	26 (28.3)	5 (25.0)	.768
: III-IV	66 (71.7)	15 (75.0)	
Duration of sedation (min) (mean, SD)	25.3 (5.5)	27.3 (7.0)	.121
Indication			.980
Cerebro-vascular accident	29 (31.5)	7 (35.0)	
Dementia	22 (23.9)	5 (25.0)	
Oral, larynx and esophageal malignancy	16 (17.4)	3 (15.0)	
Prolonged nasogastric tube insertion	8 (8.7)	1 (5.0)	
Others	17 (18.5)	4 (20.0)	

Group A: propofol-based; Group B: non- propofol based.

**Table 2:** Overall complication rate, sedation and procedure related complication, anesthetic personnel, and mortality rate (*n*, %).

	Group A	Group B	* P-*value
	(*N* = 92)	(*N* = 20)	
Overall complication rate	23 (25.0)	4 (20.0)	.636
Sedation-related complication			
Respiratory system	5 (5.4)	0	.286
Hypoxia	2 (2.2)	0	.506
Upper airway obstruction	3 (3.3)	0	.413
Cardiovascular system	17 (18.5)	4 (20.0)	.874
Hypotension	15 (16.3)	2 (10.0)	.476
Hypertension	0	1 (5.0)	.031*
Bradycardia	2 (2.2)	0	.506
Tachycardia	0	1 (5.0)	.031*
Procedure related complication			
Bleeding	1 (1.1)	0	.640
Anesthetic personnel			.986
Residents	55 (59.8)	12 (60.0)	
Anesthetic nurses	37 (40.2)	8 (40.0)	
Mortality rate	0	0	1.000

Group A: propofol-based; Group B: non-propofol based.

*Considered statistically significant.

**Table 3:** Sedative agents used in both groups.

	Group A	Group B	*P*-value
	(*N* = 92)	(*N* = 20)	
Propofol (mg/kg)			
*N* (%)	92 (100.0)	0	
Mean (SD)	0.90 (0.20)		
Fentanyl (mcg/kg)			
*N* (%)	81 (88.0)	20 (100.0)	
Mean (SD)	0.65 (0.19)	0.83 (0.23)	.018*
Midazolam (mg/kg)			
*N* (%)	74 (80.4)	18 (90.0)	
Mean (SD)	0.02 (0.01)	0.03 (0.01)	<.001*
Ketamine (mg/kg)			
*N* (%)	6 (6.5)	2 (10.0)	
Mean (SD)	0.54 (0.11)	0.79 (0.13)	.333

Group A: propofol-based; Group B: non- propofol-based.

*Considered statistically significant.

**Table 4:** Hemodynamic parameters: systolic and diastolic blood pressure (mmHg), heart rate (beat/minute) and oxygen saturation (SpO_2_, %) (mean, SD).

	Group A	Group B	* P*-value
	(*N* = 92)	(*N* = 20)	
Baseline			
SBP, DBP	137.3 (18.9), 76.6 (14.1)	139.1 (19.3), 84.6 (11.4)	.099, .585
HR, SpO_2_	73.1 (11.3), 99.3 (1.0)	79.3 (11.0), 99.8 (0.5)	.502, .267
At insertion			
SBP, DBP	119.3 (18.9), 67.7 (12.8)	124.2 (21.6), 78.5 (15.7)	.075, .436
HR, SpO_2_	70.3 (10.4), 99.8 (0.7)	76.2 (10.9), 100.0 (0.0)	.068, .618
5 minutes after insertion			
SBP, DBP	113.1 (13.5), 68.1 (12.6)	122.5 (26.3), 76.3 (16.6)	.039*, .026*
HR, SpO_2_	69.5 (11.1), 99.9 (0.6)	71.1 (16.9), 99.9 (0.2)	.226, .887
10 minutes after insertion			
SBP, DBP	107.9 (10.4), 67.7 (11.7)	121.4 (27.2), 75.1 (17.9)	.035*, .476
HR, SpO_2_	69.2 (11.7), 99.9 (0.6)	75.3 (9.8), 99.9 (0.2)	.281, .902
15 minutes after insertion			
SBP, DBP	109.9 (10.2), 68.5 (10.4)	119.9 (22.4), 74.6 (13.1)	.107, .306
HR, SpO_2_	70.7 (11.4), 99.9 (0.6)	74.1 (10.7), 100.0 (0.0)	.473, .699
20 minutes after insertion			
SBP, DBP	110.7 (11.7), 66.5 (11.9)	125.7 (20.2), 78.3 (10.7)	.091, .024*
HR, SpO_2_	70.2 (11.5), 99.9 (0.6)	73.7 (10.0), 100.0 (0.0)	.031*, .817
25 minutes after insertion			
SBP, DBP	110.1 (10.6), 70.1 (11.3)	125.2 (18.4), 79.0 (13.4)	.097, .297
HR, SpO_2_	70.7 (12.7), 99.8 (0.9)	76.5 (7.6), 100.0 (0.0)	.352, .751
30 minutes after insertion			
SBP, DBP	111.9 (10.2), 72.3 (9.4)	117.5 (13.3), 78.3 (7.1)	.516, .220
HR, SpO_2_	69.8 (14.0), 99.9 (0.3)	72.3 (5.4), 99.8 (0.5)	.394, .531

Group A: propofol-based; Group B: non-propofol based.

SBP: systolic blood pressure; DBP: Diastolic blood pressure; HR: heart rate; SpO_2_: oxygen saturation.

*Considered statistically significant.

## References

[1] Tham TCK, Taitelbaum G, Carr-Locke DL (1997). Percutaneous endoscopic gastrostomies: are they being done for the right reasons?. *QJM: An International Journal of Medicine*.

[2] Kirby DF, Delegge MH, Fleming CR (1995). American Gastroenterological Association technical review on tube feeding for enteral nutrition. *Gastroenterology*.

[3] Abuksis G, Mor M, Segal N (2000). Percutaneous endoscopic gastrostomy: high mortality rates in hospitalized patients. *American Journal of Gastroenterology*.

[4] Cohen LB, DeLegge MH, Aisenberg J (2007). AGA institute review of endoscopic sedation. *Gastroenterology*.

[5] American Society of Anesthesiologists (2002). Practice guidelines for sedation and analgesia by non-anesthesiologists: an updated report by the American Society of Anesthesiologists task force on sedation and analgesia by non-anesthesiologists. *Anesthesiology*.

[6] Larson DE, Burton DD, Schroeder KW, DiMagno EP (1987). Percutaneous endoscopic gastrostomy. indications, success, complications, and mortality in 314 consecutive patients. *Gastroenterology*.

[7] Wilson WR, Hariri SM (1995). Experience with percutaneous endoscopic gastrostomy on an otolaryngology service. *Ear, Nose and Throat Journal*.

[8] Gibson SE, Wenig BL, Watkins JL (1992). Complications of percutaneous endoscopic gastrostomy in head and neck cancer patients. *Annals of Otology, Rhinology and Laryngology*.

[9] Amann W, Mischinger HJ, Berger A (1997). Percutaneous endoscopic gastrostomy (PEG): 8 years of clinical experience in 232 patients. *Surgical Endoscopy*.

[10] McClave SA, Chang W-K (2003). Complications of enteral access. *Gastrointestinal Endoscopy*.

[11] Froehlich F, Gonvers J-J, Vader J-P, Dubois RW, Burnand B (1999). Appropriateness of gastrointestinal endoscopy: risk of complications. *Endoscopy*.

[12] Amornyotin S, Chalayonnavin W, Kongphlay S (2010). Assisted sedation for percutaneous endoscopic gastrostomy procedure in sick patients in a developing country. *Gastroenterology Insights*.

[13] Amornyotin S, Prakanrattana U, Chalayonnavin W, Kongphlay S, Kongmueng B (2009). Anesthesia for percutaneous endoscopic gastrostomy in Siriraj Hospital. *Thai Journal of Anesthesiology*.

[14] Dumortier J, Lapalus M-G, Pereira A, Lagarrigue J-P, Chavaillon A, Ponchon T (2004). Unsedated transnasal PEG placement. *Gastrointestinal Endoscopy*.

[15] Counihan T, Napolitano LM, Heard SO (1996). Transnasal insertion of percutaneous endoscopic gastrostomy in a patient with intermaxillary fixation: case report. *Journal of Trauma*.

[16] Lustberg A, Fleisher AS, Darwin PE (2001). Transnasal placement of percutaneous endoscopic gastrostomy with a pediatric endoscope in oropharyngeal obstruction. *American Journal of Gastroenterology*.

[17] Lustberg AM, Darwin PE (2002). A pilot study of transnasal percutaneous endoscopic gastrostomy. *American Journal of Gastroenterology*.

[18] Schrag SP, Sharma R, Jaik NP (2007). Complications related to Percutaneous Endoscopic Gastrostomy (PEG) tubes. A comprehensive clinical review. *Journal of Gastrointestinal and Liver Diseases*.

[19] DeLegge MH (2007). Endoscopic enteral access for enteral nutrition. *ASGE Clinical Update*.

[20] DiSario JA, Baskin WN, Brown RD (2002). Endoscopic approaches to enteral nutritional support. *Gastrointestinal Endoscopy*.

[21] Gašparović S, Rustemović N, Opačić M (2006). Clinical analysis of propofol deep sedation for 1,104 patients undergoing gastrointestinal endoscopic procedures: a three year prospective study. *World Journal of Gastroenterology*.

[22] Heuss LT, Inauen W (2004). The dawning of a new sedative: propofol in gastrointestinal endoscopy. *Digestion*.

[23] Chutkan R, Cohen J, Abedi M (2004). Training guideline for use of propofol in gastrointestinal endoscopy. *Gastrointestinal Endoscopy*.

[24] Chiu K, Yuasa K, Gambarin M (2004). Use of propofol in high risk PEG and direct PEJ placements. *Gastrointestinal Endoscopy*.

[25] Amornyotin S, Lertakayamanee N, Wongyingsinn M, Pimukmanuskit P, Chalayonnavin V (2007). The effectiveness of intravenous sedation in diagnostic upper gastrointestinal endoscopy. *Journal of the Medical Association of Thailand*.

